# Neuroinflammation in Primary Open-Angle Glaucoma

**DOI:** 10.3390/jcm9103172

**Published:** 2020-09-30

**Authors:** Stefania Vernazza, Sara Tirendi, Anna Maria Bassi, Carlo Enrico Traverso, Sergio Claudio Saccà

**Affiliations:** 1 IRCCS-Fondazione Bietti, via Livenza 3, 00198 Rome, Italy; 2 Department of Experimental Medicine (DIMES), University of Genoa, 16132 Genoa, Italy; tirendisara@gmail.com (S.T.); Anna.Maria.Bassi@unige.it (A.M.B.); 3 Inter-University Center for the Promotion of the 3Rs Principles in Teaching & Research (Centro 3R), Italy; 4 Clinica Oculistica, DiNOGMI, University of Genoa, 16132 Genoa, Italy; carloenrico.traverso@hsanmartino.it; 5 Ophthalmology Unit, IRCCS-Polyclinic San Martino Hospital, 16132 Genoa, Italy; sergio.sacca@hsanmartino.it

**Keywords:** glaucoma, trabecular meshwork defects, neuroinflammation, aging, oxidative stress

## Abstract

Primary open-angle glaucoma (POAG) is the second leading cause of irreversible blindness worldwide. Increasing evidence suggests oxidative damage and immune response defects are key factors contributing to glaucoma onset. Indeed, both the failure of the trabecular meshwork tissue in the conventional outflow pathway and the neuroinflammation process, which drives the neurodegeneration, seem to be linked to the age-related over-production of free radicals (i.e., mitochondrial dysfunction) and to oxidative stress-linked immunostimulatory signaling. Several previous studies have described a wide range of oxidative stress-related makers which are found in glaucomatous patients, including low levels of antioxidant defences, dysfunction/activation of glial cells, the activation of the NF-κB pathway and the up-regulation of pro-inflammatory cytokines, and so on. However, the intraocular pressure is still currently the only risk factor modifiable by medication or glaucoma surgery. This present review aims to summarize the multiple cellular processes, which promote different risk factors in glaucoma including aging, oxidative stress, trabecular meshwork defects, glial activation response, neurodegenerative insults, and the altered regulation of immune response.

## 1. Introduction

Glaucoma is a neurodegenerative disease characterized by the progressive loss of the retinal ganglion cells (RGCs), visual field reduction and characteristic changes in the optic nerve head (ONH) [1]. 

However, among the glaucoma types, primary open angle (POAG) is the most common. 

At present, POAG etiology, due to its multifactorial nature, is still unknown but several risk factors have been indicated as the causes of promoting its onset, namely, elevated intraocular pressure (IOP), aging, sex, ethnicity, first-degree family history of glaucoma, oxidative stress, systemic and ocular vascular factors, and autoimmunity [1,2,3,4]. Moreover, the combination of multiple risk factors is able to increase the susceptibility to glaucoma development and its severity [5]. 

Two main hypotheses are proposed, in order to explain the glaucoma pathogenesis: The mechanical and the vascular theories of glaucoma.

The mechanical theory argues that the IOP elevation, either at the lamina cribrosa or the optic nerve head (ONH) level, lead initially to hypoperfusion and then reperfusion damage [6]. Therefore, IOP elevation is considered a direct or indirect cause of RGC damage, which results in a retrograde transport blockade and the accumulation of neurotrophic factors at the lamina cribrosa instead of reaching the RGC soma. In addition to growth factor starvation, mitochondrial damage and glial cell activation, as well as oxidative stress, play an important role in promoting RGC apoptosis [7,8].

The vascular theory is based on evidence of either primary (vasospastic syndrome) or secondary vascular dysregulation found in some glaucomatous patients. The chronic impairment of ONH blood flow, which may result from an imbalance in the ocular blood flow auto-regulation and oxidative stress (vasospastic syndrome) or from systemic levels of vasoconstrictive peptides (i.e., endothelin-1), seems to be responsible for ischaemia-reperfusion nerve injury [9,10,11].

However, Saccà et al. [12] proposed a further theory in order to explain the missing link between the malfunctioning of TM cells, the IOP elevation and the loss of RGCs. In fact, the previous theories do not mention the role played by TM tissue alteration in the glaucoma cascade context.

**Figure 1:**
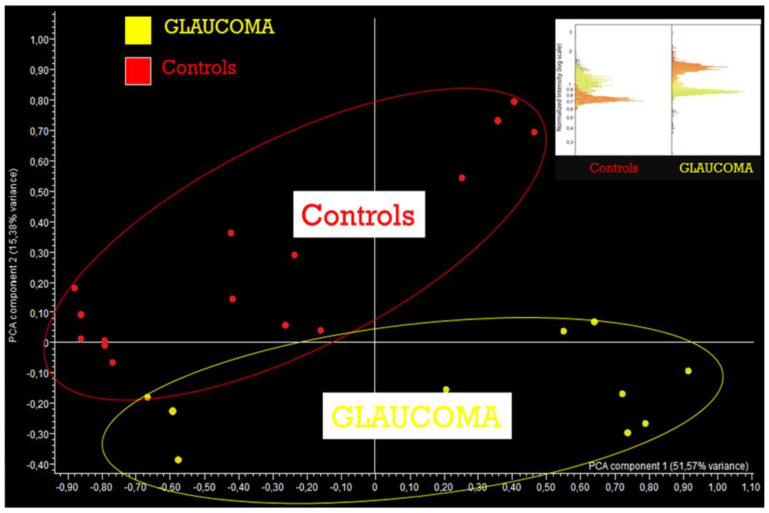
Glaucoma is a disease where pro-apoptotic signals that develop reach the head of the optic nerve promoting the death of ganglion cells. The aqueous pattern of these subjects reveals the very evident qualitative changes in proteome. This figure shows the results performed by ANTIBODY MICROARRAY in a glaucomatous AH sample. It is probable that these TM-derived proteins, released by damaged TM in the AH, may become biological signals for the retina and, in particular, for the ONH.

The alterations in the protein patterns found in the aqueous humor (AH) of POAG patients is the consequence of the progressive loss of TM cellular integrity [13,14]. Thus, these TM-derived proteins can affect both the retina and ONH behavior in the posterior segment of the eye, acting as pro-apoptotic signals for RGCs and their axons in the ONH (Figure 1). Although, it is not yet clear which of these are precisely involved in the trigger of RGC apoptosis, it is highly likely that the TM over-produced proteins, such as nestin, could represent the key molecules for glia activation or other detrimental mechanisms [12].

POAG can occur with both a normal (i.e., 15–20 mmHg) or high IOP. The first one is also known as Normal-Tension Glaucoma (NTG), while the second is called High-Tension Glaucoma (HTG). In NTG, the higher rate of hemodynamic crisis, the low systemic blood pressure, and the low ophthalmic blood pressure [15] are all thought to promote early ONH damage. However, the specific causes involved in NTG have not been discussed in this review. 

Instead, in HTG, the causes are undoubtedly associated with TM damage and an increased IOP.

As alrady known, the AH passes from the posterior chamber to the anterior chamber of the eye through two AH drainage systems, also known as the conventional and unconventional pathways of outflow [16]. Several oxidative stress-induced biochemical signals and chronic mechanical strain lead to a wide range of intracellular and extracellular morphogenic changes in the TM structure which affect its flexibility in terms of the excessive ECM deposition, the inhibition of the metalloproteinesases activity, cytoskeleton rearrangement, and so on [17]. The moment these pathological changes become chronic, there is an increase in the irreversible drainage resistance and in the elevated IOP, both of which are charateristic of HTG. 

However, through different mechanisms, both NTG and HTG are responsible for the neurodegenerative process, which begins with the early harmful responses borne by the ONH [18,19].

As far as we know, IOP remains the only modifiable risk factor (with medication or glaucoma surgery) in both types, even though it is not enough to stop RGC loss [20,21].

Several mechanisms have been suggested to promote glaucoma neurological injury, including the imbalance between neuroinflammatory and neuroprotective mediations, as well as the neurotoxicity meditated by glutamate, nitrogen oxide (NO) and oxidative stress [18,22,23,24,25]. 

This review summarizes many of the studies that have contributed to understanding the involvement of the TM in HTG and the pathological events underlying both, TM dysfunction and the POAG neurodegeneration process. 

## 2. Methods 

A systematic search of Pub Med (MEDLINE) was conducted up to and including the first part of 2020 for the preparation of this review.

Articles dealing with the pathogenetic aspects of Glaucoma, eye disease, oxidative damage and inflammation were carefully selected and reviewed. The search terms used included word combinations such as “POAG AND risk factor” (409 results); ‘‘Inflammation AND eye” (3553 results); ‘‘Neurodegeneration AND glaucoma’’(387 results); “Glaucoma AND pathogenesis AND oxidative stress‘‘ (132 results); ‘‘Nfkb AND glaucoma pathogenesis AND Nrf2” (1 result); “trabecular meshwork AND POAG” (281 results); “Neuroinflammation AND POAG” (5 results); and “long non-coding RNA AND POAG” (10 results). All abstracts were then attentively read and, if the subject was compatible with our article, the paper was reviewed in detail. This article, being a review, did not necessitate the approval from the IRCCS San Martino University Hospital (IST) Ethics Committee.

## 3. Oxidative Stress and Mitochondria Dysfunction

The increase in physiological values in the intracellular concentrations of ROS gives rise to an oxidative stress condition which can directly damage proteins, lipids, and nucleic acids. ROS are partially-reduced metabolites of molecular oxygen including superoxide anion (O^2−^), hydrogen peroxide (H_2_O_2_), hydrogen radical (OH^−^), peroxyl radical (ROO^−^), and singlet oxygen (^1^O_2_) and they can derive from both endogenous and exogenous sources. 

With aging, there is a reduction in the antioxidant network functions, which results both in oxidative damage accumulation to the cells and tissues, and a higher susceptibility to morbidity and mortality [26,27]. 

Mitochondria have been thought to contribute to aging through the accumulation of mitochondrial DNA (mtDNA) mutations and the production of reactive oxygen species (ROS). As known, human mtDNA encodes 13 polypeptide components of the respiratory chain, as well as the rRNAs and tRNAs which are necessary to support intramitochondrial protein synthesis. Therefore, both inherited mutations and somatic mtDNA mutations acquired during aging (i.e., deletions and point mutations), could contribute to several diseases including those neurodegenerative [24,25,28,29]. It has been shown that mitochondria-derived ROS may be produced by the mitochondrial matrix enzymes, the α-keto acid dehydrogenase complexes, the mitochondrial electron transport chain [30], as well as by the loss of mitochondria ability in buffering Ca^2+^ [31]. The loss of this function leads to Ca^2+^-overloaded mitochondria with a consequent formation of permeability transition pores. The opening of the permeability transition pores increases H_2_O_2_ production by a specific conformational change of complex I and, probably, also by the inhibition of the electron pathway within it, resulting in cell death via apoptosis or necrosis [32,33]. 

Therefore, the increase in oxidative stress (OS) total share is associated with several chronic diseases, including those associated with the eye (i.e., glaucoma, diabetic retinopathy and ischemic optic neuropathy) [34,35]. In particular, in POAG, the accumulation of excessive ROS can induce the trabecular meshwork damage, which results in conventional outflow pathway defects [36,37,38] and exacerbates the injury, both to the optic nerve head (ONH) and retinal ganglion cells (RGCs) [18].

In this section, the main damage induced by OS, which starts and promotes the “glaucomatous cascade”, is reported. 

### 3.1. Oxidative Stress-Related Trabecular Meshwork Damage 

The TM is the most sensitive tissue of the anterior segment of the eye to OS [39]. In the TM of glaucoma patients, significant levels of 8-oxo-2′-deoxyguanosine (8-OH-dG) [24], HSP27 and glutamine synthetase [40,41] have been found, indicating that the active oxidative agents (i.e., H_2_O_2_ and O^2−^), contained in the AH [42], were not counteracted by an adequate antioxidant defense system [43]. Moreover, both the serum and AH analysis of glaucomatous patients revealed a decrease in the total antioxidant defences [44]. In particular, Bagnis et al. [45] have reported a significant reduction in antioxidant enzymes, such as glutathione (GSH), superoxide dismutase (SOD), and glutathione S-transferase-1 enzymes, and an increase in pro-oxidant ones, including nitric oxide synthase and glutamine synthase. 

In addition, a prolonged OS condition is able to induce severe irreversible damage, such as cell death, extracellular matrix accumulation, and trabecular fusion, compromising TM functions [46,47,48,49,50]. Furthermore, the accumulation of the products of lipid peroxidation (LPO) participates in the destruction of the main tissues involved in the conventional outflow pathway [51].

The morphological analysis of glaucomatous human TM cells showed POAG-typical molecular changes, including ECM accumulation, cell death, disarrangement of the cytoskeleton, advanced senescence, NF-κB activation and the release of inflammatory markers [49,50,52]. These findings suggest that the IOP elevation, which occurs in glaucomatous patients, is related to oxidative degenerative processes affecting the human TM endothelial cells (hTMEs). Indeed, in such TM cells, ROS damage is more evident because it reduces local antioxidant activities which results in an increase of outflow resistance and in an exacerbation both of superoxide dismutase and glutathione peroxidase activities [53]. 

Furthermore, in POAG patients, mitochondrial abnormalities, such as mtDNA changes and a decrease in the mitochondrial respiratory activity are constant features, rather than gene mutations (e.g., *MYOC* and *OPTN*), which in most cases are benign [54]. Therefore, oxidative stress and a wide range of mitochondrial abnormalities (i.e., respiratory function decline, accumulation of mtDNA mutations and mitochondrial loss) are the main risk factors in POAG [29,55]. 

Chronic exposure of TM cells to oxidative stress induces several changes in the lysosomial system, which is responsible for autophagia [56], as well as cell senescence with an increase in senescence-associated-β-galactosidase [57]. In fact, oxidative stress conditions lead to lysosomal basification and the defective proteolytic activation of lysosomal enzymes with a subsequent decrease in autophagic flux and the promotion of cell senescence [12,58]. The senescence, in turn, contributes to the loss of tissue function [59] through several phenotypic changes able to alter the tissue microenvironment and to promote pathological alterations associated with aging [60]. The most important alteration found in senescent cells is the presence of a secretory phenotype, also known as the senescence-associated secretory phenotype (SASP), which contributes to tissue malfunction [61]. SASP in senescent TM cells, in fact, promotes the release of both inflammatory mediators and growth factors, affecting the function of adjacent cells, leading to a chronic activation of a stress response [62].

In addition to the senescent process, also endoplasmic reticulum (ER) defects contribute to OS-related TM damage due to its inability to take action in response to unfolded or misfolded proteins [63]. As known, ER, in order to maintain cell homeostasis, acts as the site of both the synthesis and modification of secretory proteins prior to their delivery to other secretory organelles. However, under chronic stress conditions, defects in either protein folding or defects within the ER can occur, resulting in the misfolding of specific proteins or a malfunctioning within specific ER resident proteins that are linked with several pathological states [64]. Moreover, ER stress may activate the cellular inflammatory pathway via NF-κB, induce mitochondrial changes and trigger cell apoptosis [65,66].

Saccà et al. [12] have speculated that the TM damage is responsible for both IOP elevation, which is considered an epiphenomenon, reflecting the loss of the healthy state of trabecular cells, and for the likely activation of glial cells and NMDA/AMPA receptors, through TM-derived proteins [13], in the posterior chamber of the eye [12]. 

### 3.2. Oxidative Stress and Neural Damage 

The brain is particularly susceptible to the damaging affects of ROS, which may be due to the high O_2_ uptake needed for ATP production. Large amounts of ATP are required, in order to maintain neuronal intracellular ion homoeostasis through the opening and closing of ion channels, which are involved in the action of potential propagation and neurosecretion [67]. 

As already known, the aging process is intrinsically linked to the decline of the cells’ ability to respond to oxidative damage. Therefore, the reactive oxygen and nitrogen species, produced by endogenous metabolic pathways, tend to accumulate in aging neurons rather than be effectively counteracted by the antioxidant systems [68]. 

In this regard, several previous studies have demonstrated that different neurodegenerations have common features, such as the ROS over-production, the interruption of mitochondrial function, and the failure in either the O_2_ supply or in the substrates for energy production [69,70,71]. 

In particular, the extensive damage, promoted by high Ca^2+^ traffic across the neuronal membranes and/or disruption of the ATP supply, high levels of lipid peroxidation, as well as protein carbonyl content, make up the basis for neurodegeneration onset and neuronal apoptosis [26,31,67]. 

Oxidative injury, enhanced by aging, is considered the engine of the glaucoma degenerative process and above all, the combination of both the glaucoma-derived and aging-related oxidative stress accelerate the degenerative process. The accumulation of advanced glycation end-products (AGEs), for instance, were found in the glaucomatous retina and optic nerve head [72]. Moreover, also age-related alterations, such as the glial extracellular matrix production at the optic nerve head level [73], the microglia deterioration, the cytokine and chemokine profiles, and so on, increase susceptibility to glaucomatous damage [74,75]. 

Glaucoma also has immunogenic aspects; in fact, oxidative stress-related events may alter the immune response regulation in different ways. The oxidative-induced modifications in proteins [26], lipids and DNA may activate an immunostimulatory signal to resident immune cells. In aging retina, glial cells and, in particular, the microglia are able to initiate an innate immune response [76] after inflammatory stimuli from oxidized proteins, lipids, and DNA. Therefore, low levels of oxidative stress products are effectively removed by microglial scavenger functions while the high level ones induce an over-activated state of microglia which results in an increased production of pro-inflammatory molecules (i.e., TNFα, NF-κB, nitric oxide synthase and cyclooxygenase-2) [77,78,79]. 

In addition, Tezel et al. [80] found that oxidative stress has a pathogenic role also in the retinal complement regulation. Indeed, it down-regulates the expression of complement factor H (CFH), a complement-regulatory protein which, under physiological conditions, prevents massive cell lysis and inflammation, leading to an increased vulnerability of adjacent neurons to complement-mediated injury. 

Furthermore, oxidative stress induces many downstream pathways to be triggered, which can also compromise blood barrier functions [81].

The vascular insufficiency found in some glaucoma patients is one of the possible causes that leads to low perfusion pressure, compromising ocular blood flow auto-regulation [69]. Therefore, the alterations in the blood flow modulation, which occur at the inner retina and at the superficial portion of the ONH, contribute to the reperfusion injury and ROS over-production [19]. 

The tissue hypoxia condition can develop secondary to, or independent of, an elevated IOP [18]. Under physiological conditions, an alteration in O_2_ homeostasis is quickly regulated by the hypoxia-inducible factor-1α (HIF-1α) because it acts as a hypoxia sensor though the transcription of a broad variety of genes (i.e., VEGF, iNOS, and HOMOX1) whose protein products are able to increase O_2_ delivery or facilitate the metabolic adaptation to hypoxia [82]. In glaucomatous eyes, sustained hypoxic insult or recurrent episodes of tissue hypoxia promote an increase in stressed tissue level, characterized by hypoxia, ROS generation, glial activity, immune system involvement, and/or other mechanisms yet to be identified.

In addition, oxidative stress continues to play a pivotal role in the later stage of glaucoma.

At the ONH level, the presence of nitrotyrosine (NT), also known as “the footprint of oxidative injury” [83,84], induces post-translational changes to nuclear, cytoplasmic or mitochondrial proteins [85,86,87]. In RGCs, oxidative stress is considered responsible for the presence of proteins that undergo proteolytic cleavage or exhibit post-translational modifications [88]. 

### 3.3. AH Composition in POAG Patients

The AH composition changes depending on the metabolites produced during its generation and those acquired from different anterior segment regions during its passage. Several factors may contribute to modifying these metabolites, including the time of collection, severity of the disease, body temperature and IOP fluctuations [89]. However, a constant pattern is emerging from AH analysis of glaucomatous patients in terms of significant differences in protein profiles and antioxidant defence content [90].

The increase in both endothelin 1 (ET-1) levels and NO found in AH, but not in the plasma of POAG patients [91], is probably due to the NO attempt to counteract the local agent’s activity of ET-1, thus, increasing the outflow facility of AH through NO/cyclic guanosine monophosphate [92].

Moreover, also the levels of hydroxyproline, derived from the hydrolysis of collagen [93] and acetate [94], the latter correlating with the changes in outflow dynamics, due to either cell loss or the dysfunction of sub-cellular structures, increase in POAG AH. 

Both the levels of the transforming growth factor beta 2 (TGFβ2) and plasminogen activator inhibitor-1 (PAI-1) are elevated in POAG patients [95,96,97]. Furthermore, the increase in prostaglandin H2 D-isomerase (PGDS) transthyretin (TTR) and caspase 14 in POAG AH seem to be involved in TM apoptosis [98]. 

However, the proteomic AH profile of POAG patients is completely altered compared to the healthy one. Indeed, the protein alterations detected in the glaucomatous patients were involved in the main glaucomatous pathogenic pathways, including oxidative stress, mitochondrial alterations, apoptosis, tissue disaggregation, and neuronal damage [13]. In particular, the presence of proteins, such as chains, junction proteins and cadherins which under physiological conditions contribute to tissue integrity, are the litmus test of both TM and RGC damage [99,100]. 

## 4. The NF-κB Pathway 

Nuclear factor KB (NF-κB) is one of the ubiquitous transcription factors, which play a crucial role mainly in the innate immune response. Therefore, in response to a wide range of pathogenic signals, it acts in a way to induce rapid post-translational activation, participating in cytoplasmic/nuclear signaling and regulating the gene transcriptions, which encode immunologically-relevant proteins (i.e., pro-inflammatory cytokines and secondary inflammatory mediators) [101]. Although at first, NF-κB activity has been described only in murine B lymphocytes, it has now been recognized as an important mammalian transcription factor, whose activity is regulated by the redox state of cells. In particular, the cytokines IL1β and TNFα are considered the triggers for the NF-κB signaling via their respective receptors, as well as by ligands of TLRs and certain growth factor-receptor tyrosine kinases [102,103]. Actually, the term “NF-κB” can be confusing because it is used to not only refer to the NF-κB superfamily, but also to the NF-κB subfamily and to the specific heterodimer (i.e., p50-RelA). Therefore, the NF-κB superfamily is distinguished in NF-κB proteins (p105 and p100), and Rel proteins (c-Rel, RelB, RelA/p65) [104], both sharing the Rel homology domain (RHD), a highly-conserved DNA-binding/dimerization domain. However, the members of the NF-κB subfamily are distinguished by long C-terminal domains that contain multiple copies of ankyrin repeats, which are able to inhibit Rel proteins, while the Rel subfamily contains C-terminal transactivation domains, which can activate gene transcription [105,106]. The members of the NF-κB subfamily activate the transcription only when they form dimers with the Rel subfamily members. In particular, before forming heterodimers with Rel proteins, both p100 and p105 undergo limited proteolysis which generates p52, and p50, respectively.

In the absence of stimuli, NF-κB activity is tightly regulated by the interaction with inhibitory IkB proteins in the cytoplasm. In addition, IkB proteins include several members such as IkBα, IkBβ, IkBγ and IkBε with different affinities for individual NF-κB dimers. These interactions allow the NF-κB signaling to be negatively controlled, masking both the signal of nuclear localization in the NF-κB dimer and the sequences involved in DNA binding [107]. 

However, after an intense stressful stimulus, two main pathways, namely, the canonical and the non-canonical, lead to the activation of NF-κB. The canonical pathway is the more common one of the two. It is mainly activated by pro-inflammatory cytokines (i.e., TNFa, IL-1b, IL-6, CD40L), DNA-damaging agents (camptothecin and daunomycin), Toll-like receptor (TLR) agonists and/or viruses (HTLV1, EBV) [108,109]. These stimuli, in turn, play an important role in the pathogenesis of chronic inflammatory diseases such as rheumatoid arthritis (RA), asthma, chronic obstructive pulmonary disease (COPD) and inflammatory bowel disease (IBD) [110,111,112].

Nevertheless, both pathways provide for the activation of an IkB kinase (IKK) complex consisting of the catalytic kinase subunits (IKKα and/or IKKβ), the protein NEMO (NF-κB essential modulator) and the heat shock proteins Hsp90/Cdc37 [113]. Therefore, specific phosphorylation on the IkB inhibitor determine its degradation by the 26S proteosome [114,115]. 

### 4.1. Cross-Talk among NF-κB and NRF2

NF-E2-related factor 2 (NRF2) is an important redox-sensitive transcription factor with cytoprotective role. Its activation provides for the antioxidant gene transcriptions such as NAD(P)H quinone oxidoreductase 1 (NQO1) [116,117], heme oxygenase-1 (HO-1) [118,119], glutathione S-transferase (GST), glutamate-cysteine ligase -GCS), glutathione reductase, SOD1 [120] and gluthatione peroxidase [121], by binding to the antioxidant response element (ARE) region. 

Under physiological conditions, the NRF2 activity is regulated by its repressor Keap1 (Kelch-like ECH-associated protein 1) at a cytoplasmatic level [122]. Keap1 is associated with an adaptor component of Cul3 (Cullin3)-based ubiquitin E3 ligase complex which constantly leads to the NRF2 ubiquitination and degradation as long as it remains in an inactivated state [123,124]. However, the activation of NRF2 is also controlled at a nuclear level by Bach1 and the members of SrcA chinase (Src, Fyn, Yes e Fgr), which prevent both its transcriptional activity and its nuclear accumulation [125]. 

Indeed, the phosphorilation of NRF2 on specific tyrosine residues allows for the release of NRF2 from its repressor, leading to an ARE-mediated cellular anti-oxidant response [126].

In response to OS, NRF2 is accumulated within the nucleus and heterodimerizes with Maf small protein in order to improve its binding to ARE [122]. As the nuclear activity of NRF2 ends, it is pushed out from the nucleus and it returns under the control of its inhibitors Keap1, Bach1 and SrcA chinasi [126].

A bulk of evidence has demonstrated that anti-inflammatory, anti-tumoral and phytochemical products suppress the NF-κB pathway in favor of NRF2 signaling [127,128,129] and that the MPK family modulates these mutual activation/inactivation mechanisms [130]. ROS intracellular levels are considered the molecular sensor of these above-mentioned pathways: Low levels of ROS induce the anti-oxidant gene transactivations, related to the NRF2 pathway whilst medium levels are responsible for the activation of NF-κB signaling and high levels lead to apoptosis or necrosis, due to the alteration in the mitochondrial permeability [131].

**Figure 2:**
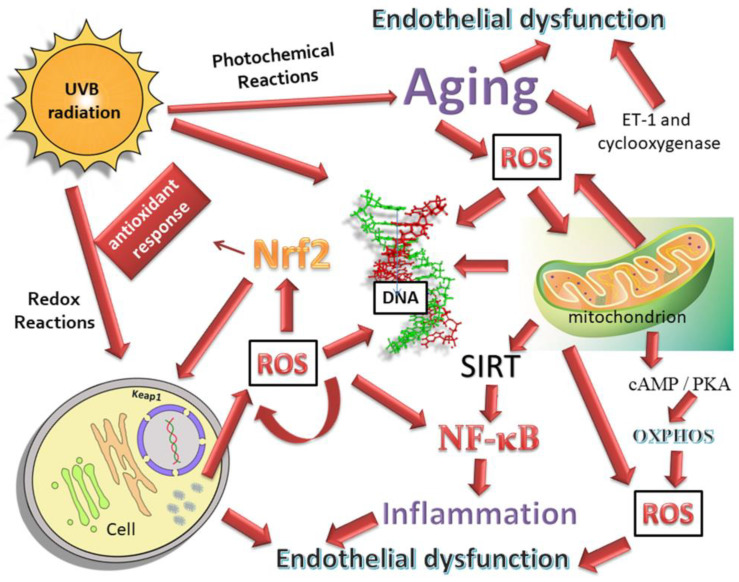
mtDNA is more susceptible to damage than nuclear DNA; mtDNA mutations have been associated with aging and neurological disorders, as well as several cancers. The main role of the mitochondria is to produce cell ATP. This process is based on OXPHOS, and ROS generation is a by-product of this process. The cAMP/PKA signaling pathway can regulate the activity of OXPHOS through its interaction with AKAP proteins in the mitochondrial membrane, while SIRT proteins, sirtuins, play a fundamental role in restoring homeostasis during stress responses. In fact, sirtuins condition the activity of the NF-κB pathways and the failure of the recovery of homeostasis causes many chronic and acute inflammatory diseases that are connected to altered glycolysis and to the oxidation of fatty acids, at least in part, by the function which is dependent on the NAD + of the sirtuins. Oxidative stress and inflammation, which contribute to endothelial dysfunction in healthy elderly subjects, are the factors, which are absolutely important for the development of glaucoma. Aging is associated with endothelial dysfunction in humans, probably linked to an elevated expression of ET-1 and cyclooxygenase.

Several molecules are seen to promote NRF2 activation. Among these, for example, the protective role of Cyclo (His-Pro) [132], has been demonstrated in a cellular rat pheocromocytoma model (PC12) in which it counteracted the paraquat toxicity via NRF2 pathway activation. Therefore, the NRF2-related anti-oxidant and anti-inflammatory enzymes have down-regulated the detriment of the NF-κB pathway (Figure 2).

### 4.2. NF-κB and TM

Chronic OS-related sub-lethal injury is involved in the pathogenesis of different diseases including atherosclerosis, glomerulonephritis, pulmonary fibrosis [133] and POAG [62]. Both ROS and NOS trigger a cascade of events that can result in cellular damage. In particular, high levels of ROS may be responsible for increased levels of nitric oxide (NO) and, consequently, for reactive peroxonitrite (ONOO-) formation [134]. 

The role of NO is well-known in both physiological conditions, in which it regulates the IOP and the ocular flow blood, and in POAG conditions, in which it is dis-regulated [135,136]. In response to inflammatory stimuli, the NF-κB activation [137] leads to the expression of the isoform Ca^2+^-independent of NO synthase (iNOS), which is an enzyme belonging to the oxidoreductases. iNOS, in turn, promotes the synthesis of NO from oxygen and arginine [138]. However, due to the pro-oxidant environment, an increase in both oxygen and nitrogen reactive species occurs, which is responsible for cellular damage, such as lipid peroxidation and MDA formation [139,140]. Moreover, iNOS over-expression can contribute to TM cell damage, and this also takes part in the OS-related cascade of events which lead to POAG [141].

There is a close relationship between the NF-κB up-regulation and the increase in pro-inflammatory cytokine production [18,142]. Indeed, in glaucoma, the NF-κB pathway regulates the glia-driven inflammatory response, the cytokine and toll-like receptor (TLR) signaling, as well as the inflammasome [143,144,145]. ROS stimulates the NF-κB activation leading to the expression of a wide range of pro-inflammatory cytokines (i.e., IL-1, IL-6 and TNF-α), which amplify the intensity of the inflammation response [146]. Moreover, in glaucomatous TM cells, endogenous IL-1 induces ELAM-1 and a pro-inflammatory cytokine expression via NF-κB [62]. 

As already known, NF-κB behaves as a damped oscillator able to synchronize itself after external stimuli with no memory [147]. Therefore, initially, the expressions of ELAM-1 and IL-1/IL-6 are very low. However, chronic NF-κB pathway stimulation boosts ELAM-1 and cytokine expressions [62,148,149,150].

NF-κB prevents cell apoptosis through the expression of genes encoding anti-apoptotic proteins [49,50,151]. TM cells from glaucomatous patients counteract the apoptotic response after oxidative stress treatment because they endogenously produce IL-1, which activates NF-κB [62]. In addition, the NF-κB activation in the early stage of glaucoma could increase the outflow facility [152], thus, promoting the matrix metalloproteinase (MMPs) expressions [49,153].

## 5. Trabecular Meshwork (TM) and Location of Outflow Resistance

The aqueous humor (AH), once secreted by the ciliary body (CB) and/or its non-pigmented epithelium, leaves the anterior eye chamber either through the conventional pathway, which involves the TM, or the uveoscleral pathway, which involves the CB [154]. In particular, the conventional pathway is recognized as being sensitive to the eye pressure because the passage of the AH is driven only by a pressure gradient [155]. Although, there is an individual susceptibility to IOP variations, both under physiological and pathological conditions, the conventional pathway has been studied for several decades due to its crucial role in glaucoma.

The Trabecular Meshwork (TM) is the anterior part of the conventional outflow pathway, located in the anterior chamber of the eye, which mainly pregulates the IOP in aqueous humor (AH) outflow and filters the AH. 

TM is a part of a complex system consisting of several components, including the Schlemm’s canal (SC), as well as the collector channels/aqueous veins. Therefore, the AH flows out through both the TM, which is the first barrier to the aqueous humor outflow, and the SC, which instead, represents the second barrier to reach the aqueous channels [42,156].

All these parts work together to maintain the IOP within a physiological range [16,157], mainly due to cellular mechano-sensory systems, mechano-transduction mechanisms, cytoskeletal responses and finely-regulated signaling pathways, as well as the interactions with the extracellular matrix (ECM) [158]. 

Anatomically, the TM is formed by connective tissue beams (lamellae) which apparently makes it look like a filter structure. Their cores consist of elastic and collagen fibers while outwardly they are covered by flat cells backed on to a basal lamina [159]. Such a particular morphology of the TM cell system increases the cell numbers exposed to the AH [14] because the latter can pass through, either the cell junctions or through TM cells that have changed their shape [160,161].

However, a non-filtering TM portion, also named the “insert region”, which is close to the Schwalbe line, exists, which probably serves as a niche for cells with adult stem-cell/progenitor properties capable of re-populating the filtering part of the TM after injury [162,163]. 

The filtering portion of the TM consists of three regions with different structures: The inner uveal meshwork (UTM), the deeper corneoscleral meshwork (CTM), and the juxtacanalicular tissue (JCT), also known as the endothelial meshwork or the cribriform region, which is localized adjacent to the inner wall endothelium of SC. 

The cells of the first two portions have a macrophage-like activity that allows for the cleaning of the AH from pigment epithelia-derived cellular debris, ROS or waste material before reaching the JCT region [163,164]. These regions protect the inner TM from these accumulations, which could interfere with resistance generation and regulation. A defective macrophage activity could be responsible for secondary form glaucoma [165].

The JCT region is defined as the point of contact between the TM endothelial cells and the SC endothelial cells (SCE) and it is also considered the crucial point for “extra” resistance in glaucomatous eyes. 

The release of chemokines and cytokines by the TM endothelial cells increases the SCE permeability [156]. 

Moreover, also the extracellular matrix (ECM) is involved in outflow resistance. Indeed, the ECM, found in the JCT region, in addition to the ECM proteins, contains matricellular proteins, such as thrombospondin-1 [166,167] and SPARC [168,169], which influence the cell function through cell-matrix interactions. Therefore, ECM homeostasis maintainance is important to avoid both the cytoskeletal manipulations of TM or SC cells and the effect on active flow pathways [170]. 

Therefore, the conventional outflow pathway is endowed with two specialized endothelial cell barriers, namely, the TME and SCE, that allow it to perform two important functions: (1) Drives the AH from the anterior chamber of the eye into the SC lumen in order to facilitate its egress; and (2) prevents the reflux of blood from the venous circulation into the anterior chamber [42]. These two barriers are attached to each other through long cell processes and they facilitate the aqueous outflow with cell-to-cell interaction mechanisms [171]. In addition, the endothelium-lined vessel of SC is distinguished in an inner and an outer wall in which endothelial cells differ for cell-specific marker expressions [172].

TME act as a control on the SCE permeability by releasing vasoactive cytokines and other factors while the SCE form ‘‘giant vacuoles’’ only when the IOP exceeds the pressure in the episcleral venous plexus. Nevertheless, the outflow resistance seems to result mainly both from a synergistic interaction through the cell-to-cell junctions between the SCE in the inner wall, their basement membrane and/or the TEM [158,173]. The endothelial cells in the inner wall, in turn, undergo a basal-apical pressure gradient due to the biomechanical micro-environment. As mentioned above, the cell’s inner walls can also be considered as an ultrastructure provided with giant vacuoles and pores, as well as F-actin arrangements (i.e., peripheral F-actin bands) [173].

Giant vacuoles, as well as intracellular and paracellular pores, are probably involved in the AH outflow resistance regulation [174]. Giant vacuoles create a small potential space between the ECM and the inner wall. Although, they appear within the cells, they are not actually intracellular structures as they derive from inner wall deformations due to the transcellular pressure drop across the cells. The wall of these invaginations is very thin but only at the point where it is at its thinnest does the intracellular pore formation occur (the so-called I-pores) [175]. Both the I-pores and the paracellular pores (B-pores), present on the inner wall of SC, contribute to the “funnelling” which is the point of the AH outflow exit. However, an increase in SC rigidity could prevent the giant vacuole formation, inhibiting I-pore formation and resulting in an IOP elevation [173,176]. In fact, in glaucomatous eyes, a significant reduction of I and B pores were found [177,178,179]. 

Therefore, the AH outflow resistance involves the entire JCT region due to either molecular changes in the TM and in its ECM composition, or to focal regions of collapse and luminal decrease within the SC, resulting in an IOP increase [180,181]. Furthermore, a chronic IOP elevation found in the HTG also leads to histopathological changes, such as a decreased SC cross-sectional area, perimeter or length. These changes result in further implications at the outer wall level such as the collapse and narrowing of collector channels (CCs), the adhesion of SCE to CC orifice walls and the blockage of CC orifices owing to the JCT herniation [182,183].

### 5.1. Local Mediators in Conventional Outflow Pathway

In the conventional outflow pathway, the resident cells use both autocrine and paracrine mediators to regulate the outflow resistance, whether positively or negatively, namely lipid-derived cytokines, nucleotides and gases [170]. 

Among the lipid-derived cytokines, the prostaglandin E2 and prostaglandin/prostamide F2a act by increasing the outflow facility in binding themselves to EP(4) or FP receptors. Therefore, the prostaglandin (PG) F2α analogs are widely used in glaucoma treatment, even though their acute and long-term effects influence both the cell behavior in the conventional pathway and the ECM turnover [184,185,186,187,188,189]. In addition, also lysophosphatidic acid and sphingosine-1-phosphate act on the AH outflow but contrary to previously stated, they decrease the outflow facility [190,191]. 

Cytokine secretions, such as interleukins (IL), interferons (IFN), colony-stimulating factors (CSF), chemokines, tumor necrosis factors (TNF), and growth factors, are implicated, either in local inflammatory processes or in non-immune functions (i.e., angiogenesis and development). However, POAG is characterized by immune activation with changes in cytokine profiles [192]. Previous studies have reported that the levels of the transforming growth factor-β_2_ (TGF-β_2_), a cytokine which may mediate the fibrotic process, were significantly increased in the AH of glaucoma patients [170,193]. In fact, in in-vitro models of human TM cells, it has been shown that the increase of TGF-β2 is responsible for an increase in the extracellular matrix deposition [194]. In addition, the increase in the oxidative stress-induced TGF-β2 activates transglutaminase 2, a ubiquitously-expressed enzyme which catalyzes irreversible post-translational modifications of proteins, forming cross-linked protein aggregates [195].

T-helper (Th) cells are considered as the main source both of pro- and anti-inflammatory cytokines. However, to better understand their function, their classification needs to be simplified. Thus, it has been considered that Th1 cells are responsible for pro-inflammatory cytokines, such as IFN-γ, IL-2, IL-12, IL-23, and TNF-alpha while Th2 cells for the production of IL-4, IL-5, IL-6, and IL-10. In fact, some studies showed that the balance of Th1/Th2 cytokines plays an important role in the damage or protection of RGCs [196,197]. In addition, other results carried out on in-vitro human TM models has revealed that long-term exposure to oxidative stress increased the levels of pro-inflammatory cytokines [198], which are responsible for TM damage and the decrease in outflow facility. Moreover, some of these cytokines secreted by TM, such as TNFα and IL-1, act in a synergistic relationship to enhance the effects of the MMP3 expression, in order to improve the TM functions [199]. Therefore, the high concentrations of IL-9, IL-12, IFN-α, IFN-γ, CXCL9 and IL-10 found in POAG AH [200] would suggest that abnormal immune environments contribute to the degeneration found in POAG. 

Small signaling molecules, such as nucleotides and nitric oxide (NO), have a role in modifying the conventional outflow. For instance, one of the cellular mechanisms which is responsible for regulating the outflow resistance is represented by TM cells and their ability to remodel the ECM. Therefore, ATP released by the TM can be converted to adenosine and then bind itself to the A1 adenosine receptors (ARs) which modulate the release of MMP2 [201,202]. Otherwise, another ATP-dependent mechanism to promote the outflow facility could derive from the binding between the TM-released ATP and the ATP-sensitive potassium channel (K_ATP_) [203].

Furthermore, the endothelial cells of the TM, which are small vessels, are regulated by both NO and ET-1 and are capable of phagocytosis and ECM production, as well as transducing signals after the stress-induced protein kinase C (PKC) attaches to the ECM [204,205,206,207]. 

Therefore, NO acts on the outflow facility by decreasing volume, contractility and/or cell-to-cell junction assembly in conventional outflow cells [170]. In particular, the TM cell volume reduction increases the intertrabecular spaces, which results in a greater cell exposure to AH [134]. In this regard, postmortem glaucomatous human eye investigations have suggested that the physiological role of NO in IOP-regulation is altered [208]. Wienderholt et al. [209] showed that the inhibition of nitric oxide synthase (NOS) causes the contraction of both the TM and ciliary muscle in-vitro. 

ET-1, unlike NO, acts by inducing the TM contraction and increasing the outflow resistance. Both, NO and ET-1 activities regulate each other with negative feed-back mechanisms.

Moreover, ET-1 induces vasoconstriction also in other parts of the eye’s anterior segment and the consequent decrease in the ocular blood flow may contribute to the RGC degeneration [134,210].

### 5.2. The Main Changes in POAG TM 

TM, as described above, is one of the main tissues involved in the conventional outflow pathway. In two forms of glaucoma, namely, HTG and primary angle-closure glaucoma (PACG), it is responsible for IOP elevation. In PACG, unlike HTG, the IOP increase results from the TM occlusion induced by the iris tissue, not directly from TM defects [43]. However, the chronic contact between the iris and the TM can cause permanent damage to the TM. 

Therefore, TM dysfunction (i.e., defect on its endothelial cells) and the reduction of its cellularity are considered the first step to the HTG onset. 

Several factors, such as oxidative stress, aging and genetic change, as well as environmental and endogenous factors are indicated as promoting TM damage. However, among the factors involved in such damage, oxidative stress is considered as the main one due to the evidence provided by both, animal and human studies [99]. 

In addition, oxidative stress could be involved in the morphological and biochemical alterations of the TM of glaucomatous eyes, due mainly to it activating both inflammatory and immune responses. In fact, chronic inflammation and oxidative stress mutually influence each other, giving rise to a vicious circle which, in turn, influences the cellular responses. 

In normal TM, the mRNA for IL-1α was undetectable while the mRNAs for IL-1β and IL-6 were found only at low levels. Cultures of human TM showed that after an exogenous stimulation such as IL1 or H_2_O_2_, the NF-κB pathway activation occurs, which results in a significant expression of the endothelial leukocyte adhesion molecule-1 (ELAM-1), IL-1α, IL-1β and IL-6 [50,62]. ELAM-1 belongs to selectin families, which are cell adhesion molecules. Such protein is produced after 2–4 h of cytokine induction and is crucial because it mediates the leukocyte-endothelial cell adhesion. The presence of ELAM-1 in POAG AH is considered a marker for the onset of TM endothelial dysfunction [211]. 

Under normal conditions, TM is prone to triggering apoptosis if the oxidant threshold is overcome. Conversely, under glaucomatous conditions, TM cells exhibit a resistance to the oxidant probably due to their endougenous IL-1 production. Therefore, it is assumed that, in these cells, the NF-κB activation, through IL-1, in spite of the chronic inflammatory activation, could promote cell survival. However, during glaucoma, a progressive loss of TM cells has been observed, thus, it is reasonable to assume that both aging and stress conditions at the basis of this disease, are over time responsible for TM cell death [99]. 

In HTG, the TM displays both chronic inflammation and tissue remodeling processes which are related to oxidative stress damage and endothelial dysfunction. Alvarado et al. [42] demonstrated that, after laser irradiation, both pro-inflammatory cytokines (i.e., IL1α, ILβ, IL8 and TNFα) and the monocytes in TME increase the permeability of SCE in order to regulate the outflow of the aqueous humor across the conventional outflow pathway. However, on one hand, the monocytes recruitment, from the blood circulation to the TM tissue, is part of a physiological mechanism sustained by the constitutive chemokines (i.e., MCP1) expressed by TM itself, on the other, the inflammatory cytokines are not and, in fact, were found either in glaucomatous human TM cells (i.e., TNFα, cIFN, IL-2, IL-3, IL-4, IL-5, IL-7, IL-12) [212,213] or in porcine/human TM cells subjected both to chronic oxidative [214] and mechanical stress [215]. Among the pro-inflammatory cytokines, IL6, IL1β and TNFα, can induce ECM remodeling and alter cytoskeletal interactions in the glaucomatous TM. 

In addition to inflammatory cytokines, also TGF-β signalling regulates the ECM turnover [216] in a pathological way. It takes part in the Extracellular Matrix Organization pathway [217] inducing elastin and collagen cross-linking enzymes, which are associated with the pathological ECM changes. In particular, TGF-β1, in cultured TM cells, is recognized as inducing the expression of smooth muscle actin (α-SMA), thus, also influencing the TM actin cytoskeleton with the cell-ECM alteration [218]. 

Furthermore, an altered or inhibited activity of matrix metalloproteinases (MMPs) are described in the POAG trabecular meshwork [219,220,221]. Under physiological conditions, MMPs are involved both in ECM turnover and cytoskeleton re-organization [222,223]. Their activity is positively regulated by PAF and/or several members of the metalloproteinase family and negatively by the tissue inhibitors of metalloprotienases (TIMPs). 

In SC and JCT of POAG eyes, for instance, the up-regulation of both MMPs (e.g., MMP1 and 3) and TIMPs has been found to probably be due to the MMP’s attempt to ECM remodelling being simultaneously counteracted by the TIMP activity [224]. Moreover, an up-regulation of MMPs also both during chronic exposure to H_2_O_2_ and post-mechanical stretching has been described [163,220,223,225].

The TM undergoes structural changes mainly due to the up-regulation of collagen, fibronectin and elastin, as well as the inhibition of MMP activities resulting in excessive ECM depositions [20,163,226,227]. Moreover, also changes, both in the glycosaminoglycans (GAGs) composition [46] and in the matricellular protein expressions (i.e., SPARC and Tenascin C), occur [168,226,228]. All these modifications result in an increased resistance of the AH outflow. The increased resistance to outflow, in turn, results in IOP elevation, also known as the main feature of HTG. 

The gap junctions in the TM play a specific role in maintaining cell-to-cell communication and allows for the passage of molecules and the electrical signal propagations [229]. The subunits, which include gap junctions, are the connexins which are integral membrane proteins. In order to form the gap junctions, one connexon hemichannel, formed by six connexins, has to bond to another connexon hemichannel present on an adjacent cell [230,231]. The mechanical stretch in HTMCs up-regulates connexin43 (Cx43) and its isoform. However, recently, also Cx26 and/or Cx31 have been suggested as being involved in an increased intercellular signalling related to the altered TM [231].

### 5.3. LncRNA-miRNA-mRNA: Role inTM and in POAG

It has been reported that long non-coding RNA (lncRNA), a RNA molecule that is not translated into a protein, could have a critical role in POAG development [232]. Indeed, lncRNA has the ability to regulate the gene expression (i.e., transcriptional interference, transcriptional activation, and chromatin modification) through its participation in the transcription regulation and gene translations. Although, the mechanisms and functions of most lncRNAs remain unclear, it has been suggested that a cross-regulation between microRNAs (miRNAs) and lncRNAs exist, which is able to favor, either the repression or expression of target mRNAs.

The mutual influence between lncRNA and miRNA is rapidly emerging and, therefore, it can act as a mRNA regulator in several manners: specific miRNAs can interact with lncRNA, in order to reduce its stability, lncRNAs may seize miRNAs favoring the expression of the repressed target mRNAs or can derepress the gene expression by competing with the miRNAs for the shared target interaction or even, some lncRNAs can produce miRNAs themselves [233]. 

For example, the lncRNA, in the anti- direction of the INK locus, single-nucleotide polymorphisms in CDKN2BAS1, has been associated with an increased risk of both POAG onset and optic nerve degeneration, probably due to its influence on the TGFβ signaling pathway or on the regulation in neighboring gene expressions [234,235,236]. 

In glaucomatous AH, three lnRNAs, such as T267384, ENST00000607393, and T342877, that can be useful for POAG diagnosis, have been identified [237]. Interestingly, ENST00000607393 has been proposed as a new therapeutic target because of its involvement in TM calcification. 

Furthermore, in an experimental model of the TM, subjected to oxidative stress, the protective role of lncRNA ANRIL has been demonstrated. Indeed, it promotes the cell survival though the down-regulation of miR-7 expression and the activation of the mTOR and MEK/ERK pathways [238]. 

Recently, a specific category of lncRNA, named competing endogenous (ce)RNAs, has also been recognized in POAG. (ce)RNAs are able to seize miRNAs, due to the presence of similar miRNA target sequences, and consequently, to favor the expression of target mRNAs, which under normal conditions, would have been repressed by these miRNA [232]. This is probably due to the alteration of post-transcriptional regulatory mechanisms of the gene expressions [239,240,241]. 

In POAG, differentially-expressed mRNAs were also found due to the above-mentioned (ce)RNA activity, especially for ubiquitin-like protein ligase, the MAPK signaling pathway [242,243], the endocytosis pathway, and the Wnt signaling pathway [244,245]. 

In addition, OIP5-AS1, which is an anti-sense lncRNA, was able to compete with several miRNAs, for example with hsa-miR-17-5p, hsa-miR-20b-5p, hsa-miR-761, hsa-miR-3619-5p, hsa-miR-24-3p, mir-27a and so on, and regulates the gene expressions [232]. Among these miRNAs, miR-27a, for instance, exerts a protective role on HTM cells under H_2_O_2_ administration [246]. Another miRNA target of OIP5-AS1, which is also involved in glaucoma, is miR-17-5p. In HTM cells, this miRNA has a role in regulating the proliferation and apoptosis in response to oxidative stress [247]. However, in POAG, the interaction between these miRNAs and OIP5-AS1 can inhibit their protective role, thus promoting cell degeneration. 

Therefore, a better understanding of this novel RNA crosstalk will lead to a significant insight into the gene regulatory networks underlying TM damage and all the tissues involved in glaucoma degeneration. 

## 6. Neurodegeneration in POAG 

Glaucoma is no longer considered a simple eye condition, but rather an optic neuropathy, characterized by a progressive degenerative disorder of the central nervous system (CNS). This knowledge comes from the fact that both retina and optic projection are part of the CNS [248,249]. 

In clinical practice, intraocular pressure (IOP) remains the only modifiable risk factor, although it is not always increased in this disease [22]. In this regard, at present, most of the results from glaucomatous neurodegeneration derive from induced or natural-occurred glaucoma animal models [250]. However, with regards to the glaucomatous neurodegeneration onset and its progression it is incorrect to make an etiological distinction about the presence or the absence of ocular hypertension. In fact, it is more accurate to consider this glaucoma outcome as a consequence of both age-related stressors and as a neurological sensitivity to pressure, regardless of its magnitude [251]. As a matter of fact, this neurodegeneration in addition to IOP, is equally favored by other concomitant factors including the increase in glutamate levels, oxidative stress, mitochondria dysfunction and low grade inflammation [252,253,254].

Moreover, neurodegeneration found in Glaucoma involves epidemiological features and mechanisms similar to that in other conditions such as Alzheimer’s (AD), Parkinson’s (PD), Amyotrophic Lateral Sclerosis (ALS), and Huntington’s disease. Indeed, despite having different etiologies, they show the same degenerative pattern (i.e., degeneration borne by axon, dendrites and cell bodies) and the specific dysregulation of Ca^2+^-dependent processes [31]. 

In particular, it has been reported that similarities exist between Glaucoma and AD in terms of the mechanisms involved in the apoptosis process of RGCs and AD cells. For example, in both conditions, β-Amyloid deposits and increased levels of tau proteins were found [255,256].

Therefore, the glaucoma neurodegenerative condition, which initially starts with axon degeneration and the loss of retinal ganglion cells (RGCs), later affects the intracranial optic nerves and the lateral geniculate nucleus (LGN), which is the first major vision center located deep within the brain, as well as in the visual cortex [257,258,259]. 

Although, not all molecular mechanisms that drive RGC death are known, it is hypothesized that RGC death could result from neurotrophic signals deprivation due to the blocking of axonal transport deriving from either disturbances in mitochondrial dynamics, which lead to apoptosis, or in the reactive oxygen species (ROS). In particular, the latter are able to act as secondary messengers and/or to modulate the protein function through the redox modifications of downstream effectors [260,261]. Therefore, the protein modifications by redox reactions increase the neuronal susceptibility to damage and glial dysfunction.

However, regardless of the causes underlying RGC death, it is assumed that for each RGC, there is one LGN neuron. Therefore, degeneration at the visual cortex level is only detectable after a loss of at least 50% of RGC [262]. Indeed, a postmortem analysis performed on LGN tissues from both glaucomatous and healthy brains showed that only in glaucomatous brains was there a reduced mitochondrial activity and a shrinkage of neurons with smaller nuclei. Moreover, in the three LGN major visual channels, which are the magnocellular, parvocellular and koniocellular pathways, a significant neural degeneration and the presence of globoid cytoplasm both in the magnocellular and parvocellular neurons and a neurochemical alteration in the koniocellular neuron were observed. In addition, a reduced cortex ribbon thickness under the calcarine sulcus and an atrophy of optic nerve tissue were found [258,263].

### 6.1. Axonal Transport Deficit 

The initial site of damage in glaucoma is at the level of the lamina cribrosa in the ONH, probably due to the axonal damage that involves a retrograde degeneration with consequent loss of the RGC somas [264]. Indeed, ONH impairment induces the mechanical blockade of the axoplasmic flow and the retrograde transport of pro-survival factors related to it, from the brain-located RGC synaptic terminal to the cell body. 

Axonal transport from cell soma to distal axon and vice versa regulates neuron homeostasis. Anterograde axonal transport is driven by kinesin and is responsible for providing both proteins and lipids of new synthesis to the distal synapse, whenever they are required and for mitochondria movement when a local energy shortage occurs.

Retrograde axonal transport is driven by dynein and it allows for both the exchange of intracellular signals over long distances and the removal of misfolded and aggregated proteins from the axon [265,266]. Moreover, the RGCs of healthy eyes receive neurotrophic factor supplies, including, for instance, the neurotrophin family of growth factors (e.g., BDNF and NGF) and the receptors to which they bind (Trk and p75NTR) both, from this transport and from the retinal Müller glia [267,268]. Therefore, it is not surprising that axonal transport defects together with the stress-signaling propagation along the axon, promote neuron decline.

Two assumptions are broadly accepted when explaining what leads to a retrograde transport defect. One assumption, agrees with considering the ONH as the point of disruption after a mechanical strain of chronic IOP increase whereas, the other indicates high trans-lamina cribrosa pressure difference responsible for the axonal damage, due to the abnormally-low cerebrospinal fluid pressure [269]. Studies carried out on rodent glaucoma models, which lack a true lamina cribrosa, showed a disrupted axonal transport at the ONH as well, suggesting that these damage mechanisms may be independent of the laminar structure [6].

Furthermore, also the trigger of astrocyte and microglia reactivity by the ONH seems to induce changes in lamina cribrosa extracellular matrix composition and oligodendrocyte death [270,271,272]. 

In addition, point mutations or small deletions in dynein heavy chain, which are the molecular motor complexes that generate force towards the minus end of microtubules, are involved in defects in retrograde axonal transport [273].

The anterograde axonal transport defect starts from the distal portion of the RGC projection out of the ONH and occurs earlier compared to that of the retrograde axonal transport one [274]. In particular, defects in kinesin-mediated anterograde transport could lead to synaptic defects and axonal decay due to an inadequate supply of new proteins and lipids from the soma to the distal synapse [273]. 

### 6.2. Microglial Activation

Microglia, together with astrocytes and oligodendrocytes, constitute the glia in the CNS. Microglia, in particular, are represented by cells with small cell bodies and ramified thin, spiny processes that surround the blood vessels and maintain a close relationship with the blood-brain barrier. Moreover, microglia are also found in the walls of large vessels and around the glial columns and cribriform plates of the lamina cribrosa. 

In the CNS, it is unlikely they participate in the vegetative maintenance of the neurons but behave as macrophage-like cells and immune surveillance cells [275]. Therefore, they are able to react to neural damage, thus further spreading the neuroinflammation process, through mechanisms which include morphological changes (amoeboid morphology), proliferation, migration and the production of inflammatory cytokines [276]. Indeed, the microglia activation enhances the neurotoxic effects by the increase in both ROS and NO levels, the release of TNFα and interleukin 6 (IL-6), and the increase in the expression of the major histocompatibility complex class I (MHC I) and II antigens [277,278,279,280,281]. 

Several previous studies have highlighted the role of TGFβ1 in regulating microglia activation and its reactivity either under basal conditions or after inflammatory stimuli [282,283,284]. In fact, brain analysis carried out on adult TGFβ1-null mice revealed an uncontrolled astroglial activation, an increase in microglia proliferation with a reduced branching of their cellular processes and strong inflammatory marker expressions (e.g., CD44) [285]. However, in animal models with neurodegeneration, despite displaying an increase in TGFβ1 amounts and their receptors, a sustained microglial activation, accompanied by neurotoxic molecule secretions, was found [286]. Interestingly, data from in vivo analysis revealed that the age-related cytotoxic microglial activation and the concomitant decrease in TGFβ1 signaling, is seen to promote the neurodegeneration [287]. 

The activation of microglia can occur after either endogenous or exogenous stimuli that are damage-associated molecular patterns (DAMPs), which include heat shock proteins (Hsp), hyaluronan, uric acid, galectins, thioredoxin (TRX), adenosine triphosphate (ATP), high mobility group box 1 (HMGB1), IL-1α and IL-33, or pathogen-associated molecular patterns (PAMPs), such as molecules released from pathogens [288,289,290,291,292]. 

DAMPs and PAMPs are recognized by specific microglial surface receptors i.e., Toll-like receptors (TLRs), NOD-like receptors (NLRs), RIG-like receptors (RLRs), AIM2-like receptors (ALRs), and C-type lectins. The activation of these receptors gives rise to the so-called inflammasome, an important molecular regulator of the inflammatory process. The activation and the assembly of the inflammasome induce the maturation of pro-inflammatory cytokines, including IL-1β and IL-18, promoting inflammation. In particular, the pro-inflammatory potential of IL-1β promotes the activation and the up-regulation of other key components of the inflammatory process, including VEGF [293], ICAM-1 [294], IL-8, CXC chemokine receptor 2 [295] and so on, which by activating neutrophils, facilitate their entry into the cornea. Moreover, the release of activated cytokines, together with the activation of caspase-1, which is a part of the inflammasome complex, can lead to pyroptosis, a type of cell death that involves the breaking open of the cells and, subsequently, intracellular component release [296]. Extracellular HSPs, for example, are indicators of cellular integrity loss and they, in turn, elicit both pro-inflammatory cytokine release (IL-1β, IL-6 and TNFα) and the innate immune response through TLR2 and TLR4. Moreover, they can also stimulate T-cell responses. 

The activation of such an inflammatory pathway leads to microglial activation, which includes typical morphological changes (e.g., the restriction of microglial cellular ramifications, and the acquisition of the typical amoeboid cell shape) and the up-regulation of microglial activation markers (e.g., MHC-I, MHC-II, CD68, CD86, and Ym1) [297,298,299]. 

However, depending on the nature of the stimulus that triggers the microglial cell activation (i.e., infections or injuries), microglial cells are distinct in two main phenotypes: The neuro-toxic phenotype, the so-called M1-like and the M2-like. Therefore, stimuli such as acute hypoxia or pressure elevation promote the M1 phenotype instead of M2 by way of NF-κB induction and TLR4 [300,301]. Indeed, the M1 phenotype is characterized by an intense inflammatory response including the release of pro-inflammatory cytokines (IL-1β, IL12, TNF-α) and inducible nitric oxide synthase (iNOS) [277,278,279,280,281], an amoeboid morphology and a high phagocytic capacity [302,303,304]. Although this response in some cases helps to remove the toxic aggregated proteins and cell debris that affect the CNS [305,306,307], such M1 activation leads to a chronic inflammatory state which, over time, is responsible for neuronal death [308,309,310,311]. 

In contrast, the second phenotype, the M2-like, plays both anti-inflammatory and neurotrophic roles that promote the neuron survival [311,312,313]. In addition, the microglial cell morphology is characterized by thin cellular bodies and ramified processes [314]. 

Although, the neural damage itself is able to activate the inflammatory response in microglia either via the bond between the nucleotides, released by damaged neurons, and the microglia purinergic receptors [315,316] or the bond between HMGB1 and the microglia CD11b receptors [317]. 

**Figure 3:**
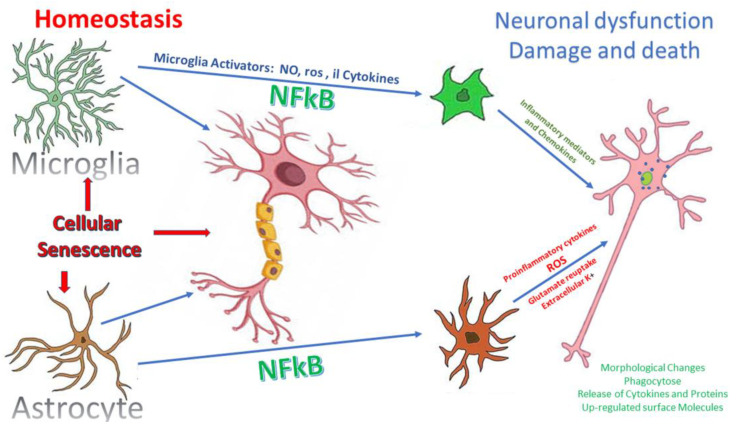
Under physiological conditions, microglia maintain the synapses and their plasticity but signaling pathways, such as NF-κB, can be activated in various ways. The activated microglia and reactive astrocytes produce ROS and neurotoxic molecules which can lead to neuronal death. In addition, the senescence of microglia and astrocytes causes inflammation and a loss of trophic support. The senescence of the oligodendrocytes reduces the myelin and influences the nerve transmission, while the senescence of the endothelial cells influences the barrier functionality. Reactive astrogliosis with up-regulation of pro-inflammatory cytokine production, excitotoxicity of glutamate and hyperexcitability of neurons may also occur.

In normal healthy human eyes, microglia are in a quiescent state [318], instead in glaucoma, both the activation of microglia and astroglia (i.e., astrocytes and Müller cells) are considered among the first events of neural damage which occurs before RGC loss. Indeed, both these retinal cell types perform immune surveillance and mediate inflammatory responses against infection, disease, or injury [314,319] (Figure 3). The correlation between both early microglial alteration and the extent of neurodegeneration has been observed in DBA/2J mice and it has also been explained that the treatment with a microglial activation inhibitor, monocycline, could slow the death of RGC [320,321].

### 6.3. Astrocytes Activation

Astrocytes are one of the cell types that belong to the glia in the CNS. Under pathological conditions, also astrocytes take part in “reactive gliosis”. Indeed, astroglial cells and the microglia work together to respond early to noxious stimuli which affect the ONH and the RGCs in glaucoma, both via the secretion of cytokines and chemokines and the activation of the adaptive immune defense response [322]. 

Astrocytes are the most abundant glia cell types in the CNS because they provide important cellular support functions for all parts of the neurons (i.e., supplying metabolites and growth factors; supporting synapse formation and plasticity; and regulating the extracellular balance of ions, fluids and neurotransmitters), and are responsible for brain homeostasis maintenance, including the axons at the lamina cribrosa and the prelaminar region of the ONH [270,323]. In addition, they form the interface between connective tissue surfaces and surround the blood vessels [270]. 

In particular, at ONH astrocyte level, two astrocyte sub-populations are found: Type1A, which expresses only glial fibrillary acidic protein (GFAP) and Type1B, which expresses both GFAP and neural cell adhesion molecule (NCAM) and appears to be responsible for ECM production in the ONH [270]. In the early stage of oxidative damage, these quiescent astrocytes become “reactive”, in order to limit the extent of injury and to promote the tissue repair process by the up-regulation of extracellular matrix components, such as collagen, proteoglycan and adhesion molecules. Therefore, an altered ECM composition leads to a loss of compliance of this dynamic tissue. The resulted glial scar, marked by an increased expression of GFAP and vimentin, in addition to representing the hallmark for CNS injury, does not support axonal regrowth [270,324]. However, in the later stages, the optic nerve atrophy suggests that this process, instead of causing a large deposition of an extracellular matrix, actually activates tissue degradation. Therefore, the exaggerated reactive astrocyte response, leads to extracellular matrix degradation though the production of a large number of neurotrophic factors and cytokines (i.e TNFα), which are able to regulate the matrix metalloproteinase (MMPs) synthesis, resulting in the typical formation of the cavernous spaces and the cupping of the ONH [271,325]. The increased MMP expression also seems to be associated with the loss of the retinal immune privilege allowing the penetration of antibodies into the eye [241]. 

Moreover, also the process of aging could be responsible for the changes in the optic nerve astrocyte function. For example, the phagocytic astrocytes in the myelination transition zone (MTZ) may become dysregulated. Thus, the up-regulation of Mac-2 in the presence of IOP elevation leads to an increase in the number of RGC with damaged axons in the retina [326]. 

In addition to the astrocyte activation at the ONH level, in glaucomatous patients, as well as in preclinical models of glaucoma, the astrocyte reactivity, found at the retina level, promotes both protective and detrimental effects [327].

Müller cells (MCs) are the major glial cells in the retina and they play a crucial role in the maintenance both of its homeostasis and its integrity thanks to the expression of several receptors related to cell growth and survival [328,329], the glutamate up-take to protect the RGC from glutamate excitotoxicity [330] and the release of nerve growth factor [330].

However, an over-activation of MCs, like microglia, promotes the release of inflammatory factors, such as tumor necrosis factor alpha (TNFα), interleukin-1 (IL-1), nitric oxide (NO) and ROS, which all contribute to exacerbate neuronal injury [331,332]. Moreover, MCs are involved in the activation of cell death receptors [333], as well as in the reduction of both potassium siphoning and water clearance [334].

In both cases, the astrocyte cell processes are connected to each other via gap junctions, of which connexins are the main components [327,335]. In particular, connexin-43 is the most abundant connexin expressed by astrocytes and, in glaucomatous patients, it is over-expressed in association with glial cell activation [336]. 

Reactive gliosis is, therefore, the mechanism involved in the neuroinflammatory response which underlies the glaucoma pathogenesis. Depending on the nature of the injury and the micro-environment at the injured site, together with the timing and the distance to the injured site [337], reactive astroglial cells can produce molecules, either with a neuroprotective role, such as IGF1 and the early induction of TNFα [338,339] or harmful molecules, such as inducible nitric oxide synthase (NOS-2), a powerful enzyme related to post-ischemic brain injury that generates excessive amounts of nitric oxide (NO) and the longer-term expression of TNFα [338,340]. 

### 6.4. Glaucoma Neuroinflammation Signaling 

In the CNS, the term “neuroinflammation” refers mainly to microglial activation but also to astrocytes response, without there being necessarily leukocyte influx from the blood [341]. However, in glaucoma the activated state of glial cells induces direct cytotoxic effects. An early up-regulation of many genes were found both in the retina and ONH, and were involved in the inflammatory pathways, such as TLRs, trasducers Trif, MyD88 as well as IL-1 and IL-6 [342,343,344,345]. 

The presence of defective immunoregulation in glaucoma has been reported [346] although there is no a direct link between the RGC loss and the aberrant humoral immunity in these patients. Indeed, a strong alteration in IgG antibody patterns (i.e., monoclonal gammopathy) in blood and AH of glaucomatous patients has been proven [347,348,349,350].

In the peripheral blood of both NTG and HTG patients, the presence of lymphocytes, that simultaneously express antigens, such as CD8 and HLA-DR or CD3 and CD8, has been found [351]. However, only in NGT patients, a significantly higher ratio between IL-2 and its soluble receptor (sIL-2R) has been reported. In fact, such an increase of sIL-2R has been previously described also in infectious, auto-immune diseases [352,353,354,355] and, interestingly, a further study has confirmed the possibility of the implication of immunological factors in glaucoma, whilst not having found a specific increase in the ratio of IL-2/sIL-2R in glaucomatous patients [196].

Furthermore, both elevated auto-antibody levels and decreased titers were found, including those against several HSPs (HSP27, HSP60, HSP70), some crystallines, structural proteins, like GFAP, vimentin and MBP, retinal deposits of immunoglobulins, and so on [4,356,357]. 

In addition, it has been reported that IOP-induced damage, both at a RGC and axon level, may persist even after IOP-lowering due to the induction of anti-HSP auto-immune responses [358]. However, since the eye is an immune-privileged site, it is not so clear why this signaling allows CD4+ T-cell infiltration in the retina to occur but it is certain that the T-cell responses are the cause of progressive neurodegeneration. 

The presence of T-cell in the retina is also supported by glial cells through signals from HSPs, oxidative stress, as well as the NF-κB pathway. Therefore, if on one hand, both HSPs and oxidative stress up-regulate the glial expression of cytokines and MHC II through TLR signaling, which in turn, stimulates the T-cell proliferation [143], on the other hand, the NF-κB activation is responsible for a secondary inflammatory cascade in the microglia and astrocytes. This amplifies the immune response with recruitment to the damaged area of other cells such as T cells [280,299,359,360]. 

Moreover, previous studies on glaucomatous patient retinas showed an up-regulation of MyD88, which is a general adaptor protein that plays an important role in the Toll/IL-1 receptor family signaling, and members of the MAPK pathway, which play an important role in several cellular programs (e.g., proliferation, differentiation, development, transformation, and apoptosis). In addition to the gene regulation mentioned above, in the ONH of DBA/2J mice, the up-regulation of 11 TLRs was also found [280,359]. 

### 6.5. Glutamate Excitotoxicity

Glutamate is an essential amino acid and is also the main fast excitatory neurotransmitter in the mammalian central nervous system (CNS). It plays an important role both in a wide variety of CNS functions (e.g., cognition, memory and learning) and in the retinal synaptic circuitry [361]. In fact, at eye level, cells such as photoreceptors, bipolar and ganglion cells are involved in the transferring of visual information from the retina to the brain through the release of glutamate.

There is a large amount of glutamate in the brain (about 5–15 mmol/kg) but only a tiny fraction of it is present at extracellular level. Indeed, glutamate stored within cells is not harmful while glutamate in the extracellular space can cause excitotoxicity via the receptor-mediated mechanisms. The extracellular fluid glutamate concentrations is physiologically around 3–4 µM, while in the cerebrospinal fluid it is about 10 µM [362,363]. 

Glutamate exerts its signaling role by acting both on ionotropic (iGluRs) and metabotropic (mGluRs) glutamate receptors and the glutamate concentration in the surrounding extracellular fluid determines the extent of receptor stimulation. 

iGluRs are homo- or heteromeric receptor protein complexes and, based on their pharmacological and electrophysiological features, they are classified into three classes: AMPA (a-amino-3-hydroxy-5-methyl-4-isoxazole propionate) receptors, kainate (2-carboxy-3-carboxymethyl-4-isopropenyle-pyrrolidine) receptors and NMDA (Nmethyl-D-aspartate) receptors. When glutamate binds iGluRs, the integral non-selective cation channels are formed [364,365].

In physiological conditions, the extracellular glutamate concentration is kept low by the glutamate uptake. This process, in turn, is regulated by glutamate transporter proteins (e.g., “High-affinity” and “Low-affinity” glutamate transporters; sodium-dependent high affinity hetero-exchange mechanisms for glutamate and ascorbate; sodium-independent and chloride-dependent high-affinity glutamate uptake), which use the electrochemical gradients across the plasma membranes as driving forces for their uptake [366,367,368,369]. 

Therefore, a high glutamate concentration, excessively activating glutamate receptors, exerts toxic effects on the cells sufficient to cause their death [362]. 

Although, the concentration of glutamate in the vitreous chamber of glaucomatous eyes has been observed to be twice that of the concentration of the vitreous chamber of healthy eyes [23], it is not known if the same result could be obtained from the extracellular space of the retinal cells of glaucomatous eyes, as this measurement has not yet been performed [370].

The glutamate toxicity may contribute to RGC death in glaucoma and, in addition, it appears to be mediated mainly by the NMDA receptor that, apart from promoting cell death, due to its greater Ca^2+^permeability, has a high affinity for glutamate and a slow inactivation [370,371,372,373]. 

Glutamate excitotoxicity, like oxidative stress, is involved in the mtDNA alteration or DNA oxidation–related mitochondrial dysfunction in retinal neurodegeneration [374]. 

Glutamate excitotoxicity over-activity in the excitatory pathway which leads to neuronal cell death through high levels of glutamate and the over-stimulation of NMDA receptors. The excitotoxic damage to RGCs could initially be promoted by either an increased glutamate synthesis or a decreased glutamate clearance [375,376]. 

The high content of extracellular glutamate and the consequent over stimulation of its receptors, induce both an increase in energy consumption because after influx of Na^+^ and Ca^2+^ they need to be pumped out again with energy expenditure [377] and the trigger of apoptosis pathways in neurons due to the immense Ca^2+^ influx [363]. The intracellular increase in the Ca^2+^ concentration leads to a depolarization of the mitochondrial membrane potential and subsequently leads to the release of various bioactive substances into the cytosol (e.g., cytochrome c), which are able to drive apoptosis through DNA fragmentation [378]. Furthermore, this excessive activation of the glutamate receptor could also cause an oxidative burst [379] and potentiate both the toxic effects of H_2_O_2_ [380] and of mutant SOD [381] which, in turn, further impairs the energy production and glutamate uptake. Therefore, it is not surprising that, as a result of energy deprivation, Na,K-ATPase inhibition, as well as early mitochondrial damage, the neurons become more vulnerable to glutamate [382,383,384]. 

So, glutamate-induced cell death can be initiated by a rapid or slow path, depending on the glutamate concentration [385]. The rapid path is characterized by acute cellular injury with a massive influx of Na^+^ and Cl^−^, with cell swelling and a necrotic cell death. Instead, the slow path is able to trigger a delayed cell death as a consequence of the initiating stimulus, and if caught early, is a reversible process [386,387]. 

## 7. Concluding Remarks

Glaucoma, still today, is considered a medical challenge due to the poor understanding of its disease mechanisms. However, several clinical and experimental studies are focusing on a wide range of cellular defects, which can drive its onset and the propagation of glaucomatous neurodegeneration. The eye undergoes constant stresses (i.e., exposure to light, to atmospheric oxygen, environmental chemicals and physical abrasion), which over time, can lead to several cell dysfunctions and ROS over-production. 

In HTG, the first step for glaucoma onset is represented by conventional pathway defects due to this being the main reason for an elevated IOP. The IOP elevation, which has been long considered the primary glaucoma risk factor, is actually nothing but epiphenomena related to the condition of both this tract and its cells. Therefore, given the high complexity of glaucoma, it is today more correct to acknowledge that many factors, including chronic inflammation (i.e., the activation of glial cells), cell dysfunctions (at TM and glia levels), mitochondrial dysfunction, oxidative stress and defects in the immune response (i.e., HSP-specific T cell responses, HSP up-regulations) all contribute to affecting the health of the cells of the eye, triggering the cascade of events that progressively leads to RGC death, optic nerve damage, and concomitant visual field loss. Although at first, NF-κB activation and the involvement of immune responses have a protective role for the eye’s homeostasis maintenance, their long-lasting activation leads to the so-called cytokine storm and detrimental effect of adaptive immune responses, which disrupt the retina homeostasis and result in the dysfunction of the immune privilege status of the eye. 

In light of these events, it is important to further explore in more detail whether the inhibition of oxidative injury can stop the vicious circle which drives glaucoma neurodegeneration. Moreover, also a better understanding of the regulatory mechanisms underlying the immune response could have a greater positive impact on glaucoma therapy. 

## Abbreviations

AC	Anterior Chamber
AH	Aqueous Humor
CB	Ciliar Body
ECM	Extra Cellular Matrix
ER	Endoplasmic Reticulum
HSPs	Heat Shock Proteins
HTG	High Tension Glaucoma
IOP	Introcular Pressure
JCT	Juxtacanalicular Connective Tissue
LC	Lamina Cribrosa
MMPs	Matrix Metalloproteinases
mtDNA	Mitochondria DNA
NO	Nitric Oxide
NTG	Normal Tension Glaucoma
ONH	Optic Nerve Head
OS	Oxidative Stress
POAG	Primary Open-Angle Glaucoma
RGCs	Retinal Ganglion Cells
ROS	Reactive Oxygen Species
SC	Schlemm’s Canal
SEM	Schlemm Endothelial Cell
Th 1/2	T-helper 1/2
TLRs	Toll Like Recepeptors
TM	Trabecular Meshwork
TME	Trabecular Endothelial Cell
